# Evaluating the Reliability and Model Fit of the 13-Item and 10-Item Big Five Inventory (Malay Version) among Malaysian Firefighters

**DOI:** 10.21315/mjms2024.31.4.15

**Published:** 2024-08-27

**Authors:** Huwaida Abdul Azis, Zairina A. Rahman, Mohd Radzniwan A. Rashid, Nizam Baharom, Hamidin Awang, Nur Hafizah Mohammad Lukman

**Affiliations:** 1 Faculty of Medicine and Health Sciences, Universiti Sains Islam Malaysia, Bandar Baru Nilai, Negeri Sembilan, Malaysia; 2 Planning and Research Division, Fire and Rescue Department of Malaysia, Negeri Sembilan, Malaysia

**Keywords:** Big Five Inventory, personality traits, firefighters, reliability test, confirmatory factor analysis

## Abstract

**Background:**

Lengthy instruments for assessing personality traits may not be applicable in certain research settings. In situations where time is scarce, a briefer measurement is preferable. However, the reliability of a briefer measurement of the Big Five Inventory (BFI) among firefighters in Malaysia has not been reported. This study aimed to investigate the reliability and model fit of the Malay version of the BFI with 13 items (BFI-13) and 10 items (BFI-10) among Malaysian firefighters.

**Methods:**

A cross-sectional study using cluster sampling was conducted in a state in Malaysia. Each respondent completed BFI-10 and BFI-13 using an online survey with a 1-month interval between each response. Reliability testing was evaluated using internal consistency and a 2-week interval test-retest. The model fit of these two BFI questionnaires was evaluated via confirmatory factor analysis (CFA).

**Results:**

A total of 124 firefighters participated in the study, with a zero-dropout rate. The Malay version of BFI-13 exhibited higher reliability by displaying good internal consistency with Cronbach’s alpha of 0.919, 0.838, 0.871 and 0.896 for the domains conscientiousness, neuroticism, agreeableness and extraversion, respectively, and acceptable test-retest reliability with moderate to good intraclass correlation (0.588–0.806). The CFA model also indicated that BFI-13 has a better model fit (comparative fit index [CFI] = 0.993; Tucker-Lewis index [TLI] = 0.991; standardised root mean squared residual [SRMR] = 0.029; root mean square error of approximation [RMSEA] = 0.035).

**Conclusion:**

The Malay version of BFI-13 is reliable and applicable enough to be supplementarily used in surveys among Malaysian firefighters. By using a brief personality assessment, it will reduce the cognitive and emotional burden on respondents.

## Introduction

Personality is a recognised subfield of psychology that describes the typical characteristics of individuals (1). Some researchers need to consider personality traits to design effective studies, accurately interpret data and make meaningful connections between individual differences and various outcomes (2). The growth in the personality field has led to multiple conceptualisations of personality (3). McCrae (4) asserted that human personality could be adequately characterised by five factors.

The majority of personality psychologists agree with McCrae that human personality is best described by five broad dimensions: i) extraversion (the tendency to be warm, sociable and assertive), ii) agreeableness (the tendency to have a pro-social orientation towards others), iii) neuroticism (the tendency to experience negative emotions such as anxiety and depression), iv) conscientiousness (the tendency to be well organised, persistent and reliable) and v) openness to experience (the tendency to be imaginative and creative) (5).

The framework of the big five personality factors served as the foundation for numerous instruments. These include the Revised NEO Personality Instrument (6), Trait Descriptive Adjective (7), International Personality Item Pool (8) and Big Five Inventory (BFI) (9). Among these, some researchers agreed that the BFI is a globally recognised instrument that is both accessible and applicable to experts outside the field of psychology (10). Thus, it is a widely recognised and extensively used survey and research instrument.

The BFI employs short phrases based on the trait adjectives that serve as prototypical markers of the BFI (5), making the items simple to comprehend. Furthermore, the BFI’s brevity does not sacrifice its good psychometric properties (11). The original version of the BFI (consisting of 44 items) has been translated and validated in various countries, including Italy, Denmark, the Netherlands, Germany and Brazil (10).

In countries such as Brazil, France, the United States and Germany, there is a shorter version of the BFI with 25 and 10 items, the performances of which have been shown to be comparable to those of the original version (12–14). The majority of the validated shortened version preserves the structure with the five factors that provide support for the original theory of the instrument (5, 10). This convergence of responses also suggests that respondents have a common understanding of the underlying construct measured by the instruments.

In Malaysia, a previous study suggested that 13 items from the original BFI are applicable to the Malaysian context (5). In the mentioned study, all the factors, except ‘openness to experience’, showed a high level of agreement among respondents in their conceptualisation of such factors. Furthermore, the validated English 10-item version (13) was translated into Malay to be used as a test instrument in the personality assessment manual of the university (15). The translation of the mentioned version was chosen owing to its good internal consistency across all domains.

In the context of surveys, there is an increasing demand to collect information in a short period of time, highlighting the importance of instruments that can be quickly completed and are reliable (10). However, the utility of the briefer BFI to measure the personalities of Malaysian firefighters remains questionable. In light of the need for a quick and reliable instrument to assess personality, BFI has the potential for use as a supplementary tool in surveys. Hence, this study aimed to examine the reliability and model fit of the translated Malay BFI-10 and BFI-13 instruments among Malaysian firefighters.

## Methods

### Study Design and Participants

This cross-sectional study involved firefighters from fire and rescue stations in the eastern state of Peninsular Malaysia. The entire population of interest was divided into distinct clusters based on their fire and rescue stations. In the present pilot study, randomised cluster sampling was employed to identify a total of five fire and rescue stations.

All eligible firefighters at each selected station were invited to participate in the study. The inclusion criteria were a work experience of more than 6 months and the ability to read and write in Malay. The exclusion criterion was a diagnosis of or receiving treatment for serious psychiatric disorders.

### Instruments

Personality traits were measured using the BFI-10 version (13) that had been translated into Malay (15) and the BFI-13 version that had been translated into Malay and then validated (5). BFI-44 reported high internal reliability, in which Cronbach’s alpha values ranged from 0.81 to 0.88, with a mean of 0.85 (16). The short versions of BFI were found to retain significant levels of reliability and validity even after reducing the items to less than one-fourth of the original BFI-44 (9).

BFI-10 consists of five subscales with two bidirectional items for each of the five major personality dimensions (13). BFI-13 has five subscales with two to three items for each of the five major personality dimensions (5). The items are rated on a 5-point Likert scale, with responses ranging from ‘strongly disagree’ to ‘strongly agree’ (17). A score between 2 and 7 is categorised as low, whereas a score between 7 and 10 is categorised as high. The higher the score, the closer is the individual characteristic to the specific domains of personality. BFI-10 and BFI-13 items were tabulated in Table 1.

### Sample Size

The sample size was determined using the computer software StatCalc (version 7.2.5.0, EPI INFO^™^ website) with a finite population size of 170 individuals. The sample size was estimated using a population proportion with a confidence level of 95% and an acceptable margin of error of 5%. The sample size was calculated by assuming the proportion of the population is unknown and anticipating maximum heterogeneity (i.e. a 50/50 split) (18). For a population proportion of 0.5 and a dropout rate of 5%, the required sample size was 124 participants.

### Data Collection

The cross-sectional study design was adopted to examine the reliability of research instruments over a brief period of time. The study was conducted from April to May 2023. The participation was entirely voluntary. The respondents were informed that their personal information would be kept confidential and strictly used for research purposes. All the respondents were briefed on the purpose and methodology of the study in the first meeting. They were reassured that there is no right or wrong answer and that the confidentiality of their answers is guaranteed.

Before conducting the research, all participants gave their informed consent. Each eligible respondent answered BFI-10 and BFI-13 separately via an online survey 1 month apart. Each instrument was completed twice for a 2-week interval of test-retest. A demographic questionnaire was attached to each tested instrument. All 124 respondents answered the questionnaires and all data were included for analysis.

### Data Entry and Analysis

IBM Statistical Packages for the Social Sciences (SPSS) version 24.0 was used to analyse the reliability testing of internal consistency and test-retest. In this investigation, test-retest reliability was estimated using the interclass correlation coefficient (ICC). For group comparisons, a value of 0.7 or higher indicates that respondent results are highly consistent (19). Internal consistency was evaluated using Cronbach’s alpha, which is generally considered as acceptable for values greater than 0.7 (20, 21). The higher the Cronbach’s alpha value, the more homogeneous is the construction of the item. Contrarily, lower Cronbach’s alpha values indicate that the constructs may contain heterogeneous factors.

IBM Analysis of Moment Structure (AMOS) version 26.0 was used to perform the confirmatory factor analysis (CFA). The CFA was used to evaluate model fit. Six fit indices were used to evaluate the model’s fit: i) discrepancy divided by degree of freedom (CMIN/df), ii) *P*-value, iii) standardised root mean squared residual (SRMR), iv) comparative fit index (CFI), v) Tucker-Lewis index (TLI) and vi) root mean square error of approximation (RMSEA).

The chi-squared test (CMIN or χ^2^) was employed to examine the hypothesis that there is a discrepancy between the model-implied covariance matrix and the original covariance matrix (22). Therefore, the insignificant discrepancy is preferred. For optimal fitting of the selected SEM, the χ^2^ test would be ideal with a *P*-value > 0.05 (23). However, χ^2^ was not presumed to be fit indices in this study due to the fact that the χ^2^ value is sensitive to sample size as a larger sample size decreases the *P*-value, where there is only a trivial misfit (22, 24). The df quantifies the number of independent values that can diverge without impeding the limitations of the model (25). At this point, CMIN/df becomes the value of interest. The model is an acceptable fit between the hypothetical model and sample data when its value is less than 3.

The CFI represents the amount of variance that has been accounted for in a covariance matrix (22). It ranges between 0.0 and 1.0. A higher CFI value indicates a better model fit. In practice, Hu and Bentler (26) suggested the CFI should be close to or greater than 0.95 to indicate a good fit. Although CFI was used to compute the data by considering the sample size, CFI was less affected by the sample size compared with the χ^2^ test (22). The TLI is a non-normed fit index that partly overcomes the disadvantages of CFI and also proposes a fit index independent of the sample size (22). A TLI of 0.90 or greater is deemed acceptable (23, 26).

RMSEA measures the difference between the observed covariance matrix per degree of freedom and the predicted covariance matrix (27). It is also referred to as a ‘badness of fit’ index, where 0 indicates a perfect fit and higher values indicate a lack of fit (26). It detects model misspecification and is less sensitive to sample size compared with the χ^2^ test. In addition, SRMR is a measure of ‘badness of fit’ because it quantifies the averaged squared differences between each bivariate empirical correlation and the respective model-implied counterpart (24). Hence, the optimal value is zero, which indicates a perfect reproduction of the empirical correlation matrix, whereas higher SRMR values reflect a poorer model fit. For a decent model fit, both SRMR and RMSEA should be less than 0.08 (23).

Moreover, acceptable factor loading and composite reliability for each item indicated that the items were contributing to the construct measurement. Acceptable factor loading should be greater than 0.5 (28). The composite reliability calculated based on factor loading indicates the consistency of the items in what they intend to measure. Composite reliability values greater than 0.7 indicate reliable factors, whereas values of 0.95 and above show unacceptable reliability because they may indicate redundancy (29). Hence, the composite reliability value should be between 0.7 and 0.95.

## Results

### Characteristics of Respondents

A total of 124 firefighters participated in the study, with a zero-dropout rate. Table 2 displays the sociodemographic characteristics of the respondents involved in the study. Out of 124 firefighters, 96.0% were males and 4.0% were females. The majority of them were married (85.5%) and finished their secondary schooling without continuing to a higher level (75.8%). Regarding their years of service, the majority have served less than 5 years (30.6%). The maximum number of years of service among respondents was 32 years. They are all of the same ethnicity (Malay) and religion (Islam).

### Reliability Analysis

The ICC of the BFI-10 ranged from 0.401 to 0.790. It indicated that this instrument has poor to good consistency. The internal consistency of each domain exhibited low internal consistency, as their Cronbach’s alpha values were less than 0.5. Besides, the values of inter-item correlation and corrected item-total correlation for all domains were in the unacceptable range. Hence, all their Cronbach’s alpha values were invalid.

The ICC of BFI-13 ranged from 0.588 to 0.806, indicating that this instrument has moderate to good consistency. Four of the five domains showed high internal consistency, as their Cronbach’s alpha values were greater than 0.7. Contrarily, the other domain (openness to experience) exhibited acceptable internal consistency, as its Cronbach’s alpha value was still greater than 0.5. Table 3 summarises the results of the BFI-10 and BFI-13 reliability analyses.

### Assessment of Model Fit

These two competing models were evaluated to determine whether the brief hypothesised five-factor model of the BFI best fits the Malaysian firefighter population. As can be seen from Table 4, only BFI-13 exhibited a non-significant discrepancy. Both BFI-10 and BFI-13 showed an acceptable fit between the hypothetical model and the sample data as their CMIN/df values were less than 3. Based on the goodness-of-fit results, BFI-13 exhibited satisfactory values for CFI and TLI, indicating a good model fit. For badness of fit, only BFI-13 had SRMR and RMSEA values less than 0.08, whereas BFI-10 had only SRMR values less than 0.08.

Based on the cutoff values, BFI-13 was chosen as the best model (χ^2^ = 63.411; df = 55; *P* = 0.204; CMIN/df = 1.153; SRMR = 0.029; CFI = 0.993; TLI = 0.991; RMSEA = 0.035) as it exhibited marginally better goodness-of-fit indices compared with BFI-10. The BFI-13 model adequately described the personality structure of Malaysian firefighters.

Despite all the aforementioned fit indices, a rough observation on the full CFA models of BFI-10 and BFI-13 showed that more than one item of BFI-10 but none of the items of BFI-13 had factor loadings of less than 0.5. The composite reliability that was calculated based on factor loading also showed that BFI-13 was more consistent in what it intended to measure. This is additional evidence that the fit of the BFI-10 model needs to be improved. As a result, BFI-13 demonstrated that its model is a better fit for data and that its usage will result in data consistency. The results of factor loading in the CFA models are summarised in Table 5.

## Discussion

Test-retest reliability is employed to measure the consistency of results when the same test is administered to the same sample at various times (30). Because the ICC of BFI-10 ranged from poor to good, the consistency of BFI-13 was better, ranging from moderate to good. Internal consistency relates to the homogeneity of questions in the same domain and their capacity to measure the same construct (30). BFI-13 exhibited an acceptable internal consistency, whereas all of the internal consistency results of BFI-10 were invalid. This finding is contrary to those of previous studies conducted in France, the United States, Germany and Brazil, where BFI-10 showed high reliability (10, 12, 13). This indicated that Asians, particularly Malaysians, might conceptualise personality traits differently compared with Europeans and Americans (31).

Fit is the capacity of a model to accurately represent the data (32). In CFA, model fit refers to how closely observed data match the relationships specified in a hypothesised model (25). A good-fitting model is one that is reasonably consistent with the data and does not necessarily require re-specification (25). Hence, CFA was employed to determine which models fit the data and which model is most plausible given the data. Although it does not confirm the veracity of the data, it will at least demonstrate the consistency of the data, as it is believed that each data point reflects how the tested population conceptualises the question (5). From the result, only the CFA model of BFI-10 contained items with factor loadings of less than 0.5 and showed weaker model fit. This suggested that BFI-10 did not have a good model fit compared with BFI-13.

There are currently no similar studies published in Malaysia to be used for comparison as a whole with this study. Nonetheless, the Malay version of BFI-13 was developed based on the reduction of research conducted among Malaysian youth (5). Contrarily, BFI-10 was translated from the result of a reduction study conducted among non-Malaysian people (13). This is probably the main reason BFI-13 exhibited higher reliability and better model fit than BFI-10. Furthermore, BFI-10 contained both positively and negatively worded items, whereas BFI-13 contained only positively worded items. Perhaps, the respondents might have been confused by the content of the reversed items. The negative wording can cause confusion when individuals are expressing their strength of agreement with those particular items (33). This result is consistent with that of a previous study from China, which also suffered from the effects associated with negatively worded items (33).

The major strength of this study is that it focused on a specific organisation. Hence, it is easier to design a procedure for respondent recruitment. Moreover, the study received good cooperation from the targeted population, as indicated by the high response rate. There was also a commendable zero-dropout rate, demonstrating the exceptional commitment and participation of all participants throughout the study duration. This may be due to the profound sense of responsibility of the firefighting workforce, which is characterised by its discipline and cohesive nature.

The limitation of this study is that it only considered briefer versions of the instrument being investigated. It is imperative to highlight that the briefer versions are indicated for use in survey contexts and not in clinical contexts. In addition, some researchers have suggested that the briefer version of the instrument does not adequately capture cultural differences in the meaning and expression of personality traits (10). To discover the best-fitting model of BFI for a certain population, it has been preferred to make a reduction as opposed to confirming the structure of other reductions. Therefore, a future study of full BFI should be executed to identify a brief BFI that is fit for Malaysian firefighters.

## Conclusion

In conclusion, the present study successfully achieved its aim of investigating the reliability and model fit of the Malay versions of BFI-13 and BFI-10 among Malaysian firefighters. The findings indicated that the BFI-13 Malay version is more reliable for the measurement of the personalities of Malaysian firefighters. Furthermore, this instrument showed better model fit, making it more useful for surveys that use personality traits as a supplementary variable in the research. These results contribute to the field by providing insights into the psychometric properties of the Malay version of BFI-13 and underscore its suitability for use in future research and survey endeavours among the target population.

## Figures and Tables

**Table 1:** BFI items used in BFI-10 and BFI-13

Instrument/Item no.^a^	Code used	Item description
BFI-10		
6	C1	*Is reserved*
22	C2	*Is generally trusting*
23	C3	*Tends to be lazy*
9	C4	*Is relaxed, handles stress well*
41	C5	*Has few artistic interests*
36	C6	*Is outgoing, sociable*
2	C7	*Tends to find fault with others*
3	C8	*Does a thorough job*
39	C9	*Gets nervous easily*
20	C10	*Has an active imagination*
BFI-13		
3	AC1	*Does a thorough job*
4	AC2	*Is depressed, blue*
7	AC3	*Is helpful and unselfish with others*
11	AC4	*Is full of energy*
13	AC5	*Is a reliable worker*
16	AC6	*Generates a lot of enthusiasm*
17	AC7	*Has a forgiving nature*
19	AC8	*Worries a lot*
28	AC9	*Perseveres until the task finished*
30	AC10	*Values artistic, aesthetic experiences*
39	AC11	*Gets nervous easily*
40	AC12	*Likes to reflect, play with ideas*
42	AC13	*Likes to cooperate with others*

Note:

a numbering based on original BFI-44

**Table 2:** Characteristics of the respondents (*n* = 124)

Characteristics	Total *n* (%)
Age	
21–27	24 (19.4)
28–34	29 (23.4)
35–41	30 (24.2)
42–49	21 (16.9)
50–56	20 (16.1)
Gender	
Male	119 (96.0)
Female	5 (4.0)
Education level	
Secondary school	94 (75.8)
Diploma/Certificate	29 (23.4)
Bachelor degree	1 (0.8)
Marital status	
Single	17 (13.7)
Married	106 (85.5)
Divorced	1 (0.8)
Years of service	
1–5	38 (30.6)
6–10	16 (12.9)
11–15	11 (8.9)
16–20	24 (19.4)
> 20	35 (28.2)

**Table 3:** Result of reliability testing on BFI-10 and BFI-13

Instrument	Factors/Items	ICC values	Cronbach’s alpha
BFI-10			
	Extraversion		
	C1	0.684	0.335^b^
	C6	0.748	
	Agreeableness		
	C2	0.401	0.295^b^
	C7	0.710	
	Conscientiousness		
	C3	0.778	0.496^b^
	C8	0.647	
	Neuroticism		
	C4	0.483	0.439^b^
	C9	0.790	
	Openness to experience		
	C5	0.560	0.141^b^
	C10	0.651	
BFI-13			
	Conscientiousness		
	AC1	0.671	0.919
	AC5	0.773	
	AC9	0.652	
	Neuroticism		
	AC2	0.695	0.838
	AC8	0.609	
	AC11	0.793	
	Agreeableness		
	AC3	0.588	0.871
	AC7	0.793	
	AC13	0.809	
	Extraversion		
	AC4	0.772	0.896
	AC6	0.747	
	Openness to experience		
	AC10	0.799	0.614
	AC12	0.664	

Note:

b invalid result due to assumption for internal consistency calculation was not met

**Table 4:** Model fit indices of CFA model

Model	*χ* *^2^*	df	*P*-value	CMIN/df	SRMR	CFI	TLI	RMSEA
BFI-10	45.81	25	0.007	1.832	0.064	0.894	0.809	0.082
BFI-13	63.41	55	0.204	1.153	0.029	0.993	0.991	0.035

Note: χ^2^ = chi-square; df = degrees of freedom; CMIN/df = normed chi-square; SRMR = standardised root mean-square residual; CFI = comparative fit index; TLI = Tucker Lewis index; RMSEA = root mean-square error of approximation

**Table 5:** Result of CFA in BFI-10 and BFI-13

Instruments	Factors/Items	Factor loading	Composite reliability
BFI-10			
	Extraversion		
	C1	0.321	0.47
	C6	0.754	
	Agreeableness		
	C2	0.348	0.30
	C7	0.497	
	Conscientiousness		
	C3	0.588	0.51
	C8	0.580	
	Neuroticism		
	C4	0.444	0.46
	C9	0.643	
	Openness to experience		
	C5	0.198	0.16
	C10	0.387	
BFI-13			
	Conscientiousness		
	AC1	0.837	0.92
	AC5	0.911	
	AC9	0.911	
	Neuroticism		
	AC2	0.785	0.84
	AC8	0.766	
	AC11	0.853	
	Agreeableness		
	AC3	0.754	0.88
	AC7	0.898	
	AC13	0.872	
	Extraversion		
	AC4	0.854	0.90
	AC6	0.954	
	Openness to experience		
	AC10	0.667	0.61
	AC12	0.665	
